# The performance of Khorana risk score for prediction of venous thromboembolism in patients with lung cancer: A retrospective cohort study

**DOI:** 10.5578/tt.202402921

**Published:** 2024-06-12

**Authors:** Deniz KIZILIRMAK, Uğur FİDAN, Yavuz HAVLUCU

**Affiliations:** 1 Department of Chest Diseases, Manisa Celal Bayar University Faculty of Medicine, Manisa, Türkiye

## Abstract

**ABSTRACT**

**
The performance of Khorana risk score for prediction of venous thromboembolism in patients with lung cancer: A retrospective cohort study
**

**Introduction:** *Cancer-related venous thromboembolism is one of the leading causes of mortality and morbidity in cancer patients. Lung cancer is the sec- ond most common cancer in the world and is closely related to venous thromboembolism. Venous thromboembolism affects survival in patients with cancer and it is important to be able to predict the possibility of thrombosis in patients with cancer. It was aimed to evaluate the predictive performance of the Khorana risk score in patients with lung cancer.
*

**Materials and Methods:** *The medical data of the patients followed up with lung cancer were analyzed retrospectively. Venous thromboembolism events in lung cancer patients were described. The relationship between the Khorana risk score and the risk of venous thromboembolism was investigated using the cumulative incidence function with compared risk models.
*

**Results:** *Eight hundred fourteen lung cancer patients were included in the study. Venous thromboembolism was detected in 79 (9.7%) of the patients. Sixty one (77.2%) of the patients had pulmonary embolism, 15 (19%) had peripheral deep vein thrombosis and three (3.8%) had venous thrombosis of other sites. The cumulative incidences of venous thromboembolism for high and intermediate Khorana risk scores were 10.1% and 9.7%, respectively (p= 0.09). The cumulative incidences of venous thromboembolism at 3, 6, 12, and
*

*
24 months were 4.7%, 5.8%, 6.4%, and 9.6% for the high-grade Khorana
*

*
risk score; 4.6%, 5.7%, 6.3% and 7.8% for the intermediate Khorana risk
*

*score (p= 0.11).*

**Conclusion:** *The Khorana risk score was not found useful in the risk stratifica- tion of venous thromboembolism (intermediate or high risk) in patients with lung cancer. New scoring systems are needed to calculate the risk of venous thromboembolism in patients with lung cancer.
*

**Key words:** *Khorana risk score; venous thromboembolism; lung cancer
*

**ÖZ**

**
Akciğer kanserli hastalarda venöz tromboembolizmin tahmininde Khorana risk skorunun performansı: Retrospektif kohort çalışma
**

**Giriş:** *Kansere bağlı venöz tromboembolizm, kanser hastalarında mortalite ve morbiditenin önde gelen nedenlerinden biridir. Akciğer kanseri dünyada en sık görülen ikinci kanser olup venöz tromboemboli ile yakından ilişkilidir. Venöz tromboembolizm kanser hasta- larında sağkalımı etkiler ve kanser hastalarında tromboz olasılığını öngörebilmek önemlidir. Bu çalışmada, akciğer kanserli hastalarda Khorana risk skorunun öngörücü performansının değerlendirilmesi amaçlandı.
*

**Materyal ve Metod:** *Akciğer kanseri tanısıyla takip edilen hastaların tıbbi verileri retrospektif olarak incelendi. Akciğer kanseri hasta- larında venöz tromboembolizm olayları tanımlandı. Khorana risk skoru ile venöz tromboembolizm riski arasındaki ilişki, karşılaştırma- lı risk modelleri ile kümülatif insidans fonksiyonu kullanılarak araştırıldı.
*

**Bulgular:** *Çalışmaya 814 akciğer kanseri hastası dahil edildi. Hastaların 79 (%9,7)’unda venöz tromboemboli tespit edildi. Hastaların 61 (%77,2)’inde pulmoner emboli, 15 (%19)’inde periferik derin ven trombozu ve üç (%3,8)’ünde diğer bölgelerde venöz tromboz vardı. Yüksek ve orta Khorana risk skorları için kümülatif venöz tromboembolizm insidansı sırasıyla %10,1 ve %9,7 idi (p= 0,09). Üç, 6, 12 ve 24. aylarda kümülatif venöz tromboembolizm insidansları, yüksek dereceli Khorana risk skoru için %4,7, %5,8, %6,4 ve
*

*
%9,6 idi; orta Khorana risk skoru için %4,6, %5,7, %6,3 ve %7,8 (p= 0,11).
*

**Sonuç:** *Khorana risk skoru, akciğer kanserli hastalarda venöz tromboemboli (orta veya yüksek risk) risk sınıflandırmasında yararlı bulunmadı. Akciğer kanserli hastalarda venöz tromboembolizm riskini hesaplamak için yeni skorlama sistemlerine ihtiyaç vardır.
*

**Anahtar kelimeler:** *Khorana risk skoru; venöz tromboembolizm; akciğer kanseri
*

## INTRODUCTION

The annual mean incidence of venous thromboem- bolism (VTE) is between 39-115/100.000 (1). VTE related mortality increases depending on co-morbid- ities, the presence of pulmonary thromboembolism and the severity of the disease. The 30-day mortality due to pulmonary thromboembolism was found to be 2.3% in the low-risk group and 11.4% in the high- risk group (2). Among the risk factors that increase the susceptibility to VTE are many factors such as a his- tory of VTE, major trauma, surgical operation, recent hospitalization, long flights, immobility, obesity, and concomitant heart diseases (3,4).

The presence of cancer also poses a risk for the development of VTE. VTE might occur in 4% to 15% of cancer patients (5). VTE development is more common in advanced cancer patients (6). The development of VTE in cancer patients directly affects mortality. Patients with lung cancer who develop VTE in the early stages have a worse survival rate than patients who do not develop VTE, even after evaluation in terms of cancer stage, comorbidities, and performance status (7). Considering that VTE affects survival in patients with cancer, it is particularly important to be able to detect the possibility of thrombosis in advance.

Since VTE complication is frequently seen during chemotherapy, it is recommended to calculate the risk before initiating chemotherapy (8). There is a need for risk scoring systems that can assist the

clinician in the evaluation of individuals with cancer in order to diagnose VTE correctly. Many scoring systems have been developed in the world to determine the risk of VTE in patients with cancer, and the most accepted one among them is the scoring system proposed by Khorana et al. in 2008 (9). The Khorana risk score is recommended by the most recently updated guidelines of the American Society of Clinical Oncology (ASCO) and the National Comprehensive Cancer Network (NCCN) to select outpatient cancer patients who require thromboprophylaxis (10,11). Early and accurate diagnoses can be made to prevent mortality and morbidity by using the Khorana risk score and similar predictive scoring systems. In addition, unnecessary examinations can be prevented and costs can be reduced.

Five different parameters were observed in the Khorana risk score as the number of platelets before chemotherapy, the number of leukocytes before chemotherapy, the amount of hemoglobin before chemotherapy, the organ of cancer and body mass index. According to these evaluations, the risk of developing VTE in patients was divided into three different categories as low, intermediate and high risk (9). Lung cancer is an important public health problem as it causes the most deaths among all cancer types. Predicting VTE risk in lung cancer patients classified as intermediate or high-risk according to the Khorana risk scoring is important for reducing mortality and morbidity. In this study, it was

aimed to investigate the prediction performance of the Khorana risk score in lung cancer patients in the intermediate and high-risk groups.

## MATERIALS and METHODS

The study included patients in Manisa Celal Bayar University Chest Diseases Department who had been diagnosed with lung cancer within the 10 years prior to the study (July 2010 to June 2020). In this retro- spective cohort study, the incidence of VTE in lung cancer patients was investigated. Participants in the study were to be older than 18 years of age and diag- nosed with pathologically proven lung cancer. Those who had missing file data, had genetic thrombophil- ia, had irregular hospital follow-ups, dropped out of follow-up or started to be followed in another center were excluded from the study. In addition, patients who received venous thromboembolism prophylaxis during the follow-up period and patients who had surgery, interventional procedures or any other trig- gered reason were excluded from the study, as this would affect the risk of developing venous thrombo- embolism. Ethical approval for the study was obtained from the Health Sciences Ethics Committee.

The follow-up files of the patients who were followed up with the diagnosis of lung cancer during the scheduled date of the study were reviewed retrospectively. Patient demographics such as age, gender and body mass index (BMI) and also medical information such as lung cancer type, disease stage, treatments and comorbidities were recorded. VTE events of patients in the study were calculated retrospectively by investigators who were unaware of the clinical prediction score being calculated.

Khorana risk score parameters of all patients were calculated and recorded retrospectively. Patients were divided into three categories according to the probability of developing thromboembolism as low, intermediate and high risk using five different parameters in the risk scoring model developed by Khorana et al. In the evaluation; the patients were divided into groups by scoring the primary cancer region (very high risk: Stomach, pancreas 2 points, high risk: Lung, lymphoma, gynecological, bladder, testis: 1 point), pre-chemotherapy platelet count (>350.000/mL: 1 point), pre-chemotherapy hemoglobin level (<10 mg/dL: 1 point), pre- chemotherapy leukocyte count (>11.000/mm^3^: 1 point) and body mass index (>35 kg/m^2^: 1 point). According to the Khorana risk score, patients with a

score of >2 are considered high-risk, those with a score of 1-2 are considered intermediate-risk, and those with a score of 0 are considered low-risk (9).

Patients with lung cancer are in the intermediate or high-risk group because of their diagnosis when Khorana risk assessment is performed on. Since they would be at least in the intermediate risk group, the patients were examined in two groups as intermediate risk and high risk. Patients in the two groups were compared for the cumulative incidence rate of VTE events at 3, 6, 12, and 24 months. The data from the study were statistically analyzed using the “SPSS Statistics 21” program. Descriptive data included frequency, percentages, median (interquartile range), mean, and standard deviation values. The numerical variables used in the comparisons followed a normal distribution. The conformity of the variables to the normal distribution was evaluated with the Shaphiro- Wilk test. The independent sample T-test was applied to analyze these variables. The Chi-square test was used for comparing categorical variables. To identify the variables that influence mortality, comparative correlation analyzes (Pearson) were used. A p< 0.05 value was considered significant in statistical evaluations. The relationship between Khorana risk score and VTE was analyzed with the competitive risk models of Fine and Gray using the cumulative incidence function (12).

## RESULTS

The follow-up files of a total of 912 lung cancer patients followed between July 2010 and June 2020 were reviewed. A total of 98 patients were excluded from the study because 36 patients had missing file data, one patient had genetic thrombophilia, 29 patients had irregular hospital follow-ups, and 32 patients were out of follow-up. Eight hundred four- teen patients were identified as the study group (Figure 1). VTE was detected in 79 (9.7%) of 814 patients. Sixty one (77.2%) of the patients with VTE had pulmonary embolism, 15 (19%) had peripheral deep vein thrombosis, and three (3.8%) had throm- bosis at other sites.

The patients included in the study had a mean age of

61.9 ± 9.2. Seven hundred thirty six (90.4%) were male and 78 (9.6%) were female. The mean BMI was

25.3 ± 8.5. Three hundred ten eighty (39.1%) of the patients had a history of chronic obstructive pulmonary disease (COPD), 72 (8.8%) diabetes mellitus, 62 (7.6%) chronic kidney disease, 53

**Figure 1.** Consort diagram.

(7.6%) other organ malignancies, 367 (45.1%)

ischemic heart disease, 74 (9.1%) neurological disease, and 13 (1.6%) rheumatologic disease when the additional comorbidities of the patients were examined (Table 1).

Two hundred fifty nine patients (31.8%) had small cell lung cancer. One hundred six (40.9%) of these patients were in the advanced stage. Number of patients with non-small cell lung cancer were 555 (68.2%). When classified according to their stages, 10 (1.8%) patients were in stage 1, 26 (4.7%) patients were in stage 2, 105 (18.9%) patients were in stage 3A, 274 (49.4%) patients were in stage 3B and 140 (25.2%) patients were in stage 4. When the patients

were classified according to the treatment options, 32 (3.9%) patients received surgery, 254 (31.2%) patients

received chemotherapy, 83 (10.2%) patients received

radiotherapy, 354 (43.5%) patients received chemotherapy and radiotherapy combination, and 91 (11.2%) patients received palliative therapy (Table 2).

Number of patients with body mass index >35 kg/m^2^ was 59 (7.2%), patients with hemoglobin value <10 mg/dL was 83 (10.2%), patients with white cell count (WBC)> 11.000 x 10^6^/L was 146 (17.9%), and

patients with platelet count> 350.000 x 10^6^/L was

157 (19.3%). Patients with an intermediate risk Khorana score (1-2) were 658 (80.8%) and patients with a high Khorana score (≥3) were 156 (19.2%) (Table 3).

Total cumulative incidences of VTE for high and intermediate Khorana risk scores were 10.1% [95% confidence interval (CI), 7.8-19.2 and 9.7% (95% CI, 7.2-15.3)], respectively. The difference was not

statistically significant (p= 0.09). The cumulative incidences of VTE at 3, 6, 12, and 24 months were

4.7%, 5.8%, 6.4%, and 9.6% for the high Khorana

risk score and 4.6%, 5.7%, 6.3%, and 7.8% for the intermediate Khorana risk score. These differences were not statistically significant (p= 0.11).

## DISCUSSION

Although the Khorana risk score is a widely used scoring system to determine the risk of VTE in cancer patients, it was found ineffective in determining the risk of VTE in patients with lung cancer in the intermediate and high-risk group in our study. Study findings concluded that the Khorana risk score was ineffective in predicting VTE events at 24-month follow-up in retrospectively followed up lung cancer patients.

Lung cancer patients comprised 554 (20.5%) of the derivation cohort group and 236 (17.3%) of the validation cohort group in the study in which the Khorana risk score was presented. When all patients were evaluated in terms of stage, only 36.9% of the derivation cohort group and 34.9% of the validation cohort group were stage four cancer patients (9). In our study, monitoring of the results of 814 lung cancer patients, the fact that lung cancer was observed at an older age compared to other cancer types and its more aggressive course may explain the ineffectiveness of the Khorana risk scoring in this group.

Similar to our study, there are numerous studies supporting that the Khorana risk score is not effective in identifying high-risk patients in patients with lung cancer (13-15). In the lung cancer database study by Mansfield et al., the cumulative risk of VTE in patients with lung cancer was similar in the intermediate and high-risk groups according to the Khorana risk score. However, according to the results of this study, being in the high-risk group was a mortality predictor (13). In a meta-analysis study examining 3293 cancer patients, the Khorana risk score was found to be ineffective in lung cancer, although it was effective in determining the risk of VTE in other types of cancer (15).

When the studies on the effectiveness of the Khorana risk score were examined, the negative predictive value was of the test 98.5% and the positive predictive value was 7.1% in high-risk patients. With these results, it is seen that it is mainly effective in

identifying patients with low risk for VTE (9). The fact that the Khorana risk score was not found to be effective in this patient group coincides with the low positive predictive value of the scoring, since the patients with lung cancer are at least in the intermediate risk group due to the location of the cancer.

There are also studies investigating the modified Khorana risk score, which examines the d-dimer level in addition to the Khorana risk score parameters in order to evaluate the risk of developing VTE in lung cancer patients. Since D-dimer level is affected by factors such as tumor, infection, trauma and surgery, its accuracy is low when evaluated alone. Modified Khorana risk score, which combines D-dimer level with Khorana risk score, was found to be effective in identifying high-risk patients for VTE in patients with lung cancer (16).

The main limitations of this study are that it is a single-centered study, staging was not done according to the 8th TNM classification because it was conducted

retrospectively, the patients could not be classified in terms of the treatment agents they received, and it was designed retrospectively. Another limitation of the study is that due to its retrospective cohort design, all risk factors associated with venous thromboembolism could not be evaluated in detail. In addition, the risk status of patients in terms of mortality was not investigated in this study. The strengths of this study are that it includes data of patients followed for two years in the same center, it is a cohort study, it evaluates the lung cancer patient group alone and all patients could be evaluated in terms of Khorana risk score.

## CONCLUSION

VTE is an important cause of mortality and morbidity in patients with lung cancer. In the evaluation of these patients in terms of VTE risk, investigation of new scoring systems such as modified Khorana risk scoring and prospective studies on the effectiveness of Khorana risk scoring may be useful.

In terms of Khorana risk score, the incidences of VTE at 3, 6, 12 and 24 months of lung cancer patients in the intermediate and high-risk groups were similar. Khorana risk score was not found useful in VTE risk stratification in lung cancer patients. There is a need to develop new scoring systems to calculate the risk of VTE in patients with lung cancer.

**Ethical Committee Approval:** This study was approved by the Manisa Celal Bayar University Health Sciences Ethics Committee (Decision no: 20.478.486/1042, Date: 01.12.2021).

## CONFLICT of INTEREST

The authors declare that they have no conflict of interest.

## AUTHORSHIP CONTRIBUTIONS

Concept/Design: DK, YH Analysis/Interpretation: DK, YH Data acqusition: UF, DK Writing: All of authors

Clinical Revision: YH, DK Final Approval: All of authors

## REFERENCES

1 *
  Wendelboe AM, Raskob GE. Global burden of thrombo- sis: Epidemiologic aspects. Circ Res 2016; 118: 1340-7. https://doi.org/10.1161/CIRCRESAHA.115.306841*
2 *
  Elias A, Mallett S, Daoud-Elias M, Poggi JN, Clarke M. Prognostic models in acute pulmonary embolism: A sys- tematic review and meta-analysis. BMJ Open 2016; 6: e010324. https://doi.org/10.1136/bmjop-en-2015-010324*
3 *
  Rogers MA, Levine DA, Blumberg N, Flanders SA, Chopra V, Langa KM. Triggers of hospitalization for venous throm- boembolism. Circulation 2012; 125: 2092-9. https://doi.org/10.1161/CIRCULATIONAHA.111.084467*
4 *
  Anderson FA Jr, Spencer FA. Risk factors for venous throm- boembolism. Circulation 2003; 107: I9-I16. https://doi.org/10.1161/01.CIR.0000078469.07362.E6*
4 *
  Falanga A, Zacharski L. Deep vein thrombosis in cancer: The scale of the problem and approaches to management. Ann Oncol 2005; 16: 670-96. https://doi.org/10.1093/annonc/mdi165*
5 *
  Sørensen HT, Mellemkjaer L, Olsen JH, Baron JA. Prognosis of cancers associated with venous thromboembolism. N Engl J Med 2000; 343: 1846-50. https://doi.org/10.1056/NEJM200012213432504*
6 *
  Kourelis TV, Wysokinska EM, Wang Y, Yang P, Mansfield AS, Tafur AJ. Early venous thromboembolic events are associ- ated with worse prognosis in patients with lung cancer. Lung Cancer 2014; 86: 358-62. https://doi.org/10.1016/j.lungcan.2014.10.003*
7 *
  Lyman GH, Khorana AA, Kuderer NM, Lee AY, Arcelus JI, Balaban EP, et al. Venous thromboembolism prophylaxis and treatment in patients with cancer: American Society of Clinical Oncology clinical practice guideline update. J Clin Oncol 2013; 31: 2189-204. https://doi.org/10.1200/JCO.2013.49.1118*
8 *
  Khorana A, Kuderer NM, Culakova E, Lyman GH, Francis CW. Development and validation of a predictive model for chemotherapy-associated thrombosis. Blood 2008; 111(10): 4902-7. https://doi.org/10.1182/blood-2007-10-116327*
9 *
  Lyman GH, Bohlke K, Khorana AA, Kuderer NM, Lee AY, Arcelus JI, et al. Venous thromboembolism prophylaxis and treatment in patients with cancer: American Society of Clinical Oncology clinical practice guideline update 2014. J Clin Oncol. 2015; 33(6): 654-6. https://doi.org/10.1200/JCO.2014.59.7351*
10 *
  Streiff MB, Holmstrom B, Ashrani A, Bockenstedt PL, Chesney C, Eby C, et al. Cancer-associated venous throm- boembolic disease, version 1.2015: Featured updates to the NCCN Guidelines. JNCCN J Natl Compr Cancer Netw 2015; 13(9): 1079-95. https://doi.org/10.6004/jnccn.2015.0133*
11 *
  Fine JP, Gray RJ. A proportional hazards model for the subdistribution of a competing risk. J Am Stat Assoc 1999; 94: 496-509. https://doi.org/10.1080/01621459.1999.10474144*
12 *
  Mansfield AS, Tafur AJ, Wang CE, Kourelis TV, Wysokinska EM, Yang P. Predictors of active cancer thromboembolic outcomes: Validation of the Khorana score among patients with lung cancer. J Thromb Haemost 2016; 14(9): 1773- 8. https://doi.org/10.1111/jth.13378*
13 *
  Di Nisio M, van Es N, Rotunno L, Anzoletti N, Falcone L, De Tursi M, et al. Long-term performance of risk scores for venous thromboembolism in ambulatory cancer patients. J Thromb Thrombolysis 2019; 48(1): 125-33. https://doi.org/10.1007/s11239-019-01845-6*
14 *
  van Es N, Ventresca M, Di Nisio M, Zhou Q, Noble S, Crowther M, et al. The Khorana score for prediction of venous thromboembolism in cancer patients: An individ- ual patient data meta‐analysis. J Thromb Haemost 2020; 18(8): 1940-51. https://doi.org/10.1111/jth.14824*
15 *
  Li S, Gao P, Qiu J, He X, Mao Y. A modified Khorana score as a risk assessment tool for predicting venous thrombo- embolism in newly diagnosed advanced lung cancer. J Thromb Thrombolysis 2021; 52(3): 898-903. https://doi.org/10.1007/s11239-021-02396-5*

